# Body composition indices in Brazilian adults: age-specific and sex-specific percentile curves

**DOI:** 10.20945/2359-3997000000124

**Published:** 2019-04-15

**Authors:** Cristiane Junqueira de Carvalho, Giana Zarbato Longo, Leidjaira Lopes Juvanhol, Adriana Maria Kakehasi, Patrícia Feliciano Pereira, Kátia Josiany Segheto, Brunnella Alcântara Chagas de Freitas, Andréia Queiroz Ribeiro

**Affiliations:** 1 Universidade Federal de Viçosa Departamento de Medicina e Enfermagem Universidade Federal de Viçosa Viçosa MG Brasil Departamento de Medicina e Enfermagem, Universidade Federal de Viçosa (UFV), Viçosa, MG, Brasil; 2 Universidade Federal de Viçosa Programa de Pós-Graduação em Ciência da Nutrição Departamento de Nutrição e Saúde Universidade Federal de Viçosa Viçosa MG Brasil Programa de Pós-Graduação em Ciência da Nutrição, Departamento de Nutrição e Saúde, Universidade Federal de Viçosa (UFV), Viçosa, MG, Brasil; 3 Universidade Federal de Minas Gerais Departamento do Aparelho Locomotor Faculdade de Medicina Universidade Federal de Minas Gerais Belo Horizonte MG Brasil Departamento do Aparelho Locomotor, Faculdade de Medicina, Universidade Federal de Minas Gerais (UFMG), Belo Horizonte, MG, Brasil

**Keywords:** Body composition, DXA scan, sarcopenia, skeletal mass index

## Abstract

**Objective:**

To establish percentile curves for measures and indices of body composition by age and sex and compare them with data from other ethnic groups.

**Subjects and methods:**

Cross-sectional, population-based study with adults aged 20-59 years (n = 689). Percentile curves adjusted by a third degree polynomial function were constructed for skeletal mass index (SMI), fat mass index, body fat, and load-capacity metabolic indices (LCMI) based on dual-energy X-ray absorptiometry (DXA).

**Results:**

SMI_weight_ and SMI_BMI_ showed decline from the third decade of life in both sexes, whereas SMI_height_ was not able to identify lean mass loss over the ages studied. There was a slight drop at the end of the fifth decade (50-59 years) in men. Among Americans and Chinese, the 50th percentile curve of SMI_height_ showed an earlier decline. The estimates of adiposity and LCMI curves peaked between 40-49 years and Americans and Chinese maintained an upward curve throughout adulthood.

**Conclusion:**

The data and curves showed that the SMI adjusted for BMI and body weight were more adequate in detecting the decline of lean mass in adults due to aging. In contrast, SMI_height_ had a positive correlation with age and its curve increased throughout the evaluated age groups. The results contribute to the evaluation to the nutritional status of adults and to the prevention and treatment of outcomes related to adiposity and deficit lean mass.

## INTRODUCTION

Body composition evaluation is an important tool to assess the nutritional status and health of the population, especially regarding the prevention and treatment of cardiometabolic diseases (1). Despite being a non-invasive, low-cost, and reproducible method, the body mass index (BMI) has limitations, since it can not discriminate between fat mass and lean mass. Thus, using only BMI to measure body composition may be inappropriate because it groups people with different degrees of adiposity and lean mass into the same category of cardiovascular risk (1-4). In this sense, dual-energy X-ray absorptiometry (DXA) enabled us to advance in the concept of body composition and its relationship with health, as it allows the estimation of adiposity and lean body mass regardless of the nutritional status established by BMI (5).

Lean mass, whose main component is skeletal muscle mass, changes throughout the lifespan and research shows a higher prevalence of muscle mass deficit after the age of 60 years (5-9). At the end of the 1990s, Baumgartner proposed an index for evaluation of low lean mass in the elderly and studied its association with falls, fractures, functional disability, and frailty, introducing the term sarcopenia (8). Since then, new lean mass indices have been developed and studies found associations between low lean mass and other health outcomes which were not related to the musculoskeletal system such as cardiometabolic diseases (6,10-12).

In addition to the index proposed by Baumgartner and cols*.* (8), which relates appendicular lean mass to height, other body composition indices assess the contribution of lean mass and adiposity to predict health risk. Among these, we highlight those indices that relate appendicular lean mass to body weight (11) and to BMI (10); total fat mass to height (13) and to total fat free mass (2); and, finally, the one that relates fat mass of the trunk to the appendicular lean mass (2). The latter two are termed load-capacity metabolic indices (2).

Reference data for lean mass and adiposity, as well as estimates of body composition indices using DXA are available for specific populations, which limits their generalization to other groups. In addition, the emergence of a new research topic associating lean mass deficit with cardiometabolic outcomes increased the interest in lean mass in adults, since, for years, discussions of the clinical implications involved only the elderly population (14-17). Thus, the development of reference values according to age and sex in different populations, especially among adults, has become fundamental (15,18-21).

Therefore, the objective of the present study was to establish percentile curves for measures and indices of body composition, specific for age and sex, from the adult population of a Brazilian city and to compare them with data from other ethnic groups. It should be noted that, to the best of our knowledge, this is the first population-based study to produce reference values among Brazilian adults.

## SUBJECTS AND METHODS

### Design and study population

This cross-sectional population-based study was carried out in the urban area of the city of Viçosa-MG, with adults (20-59 years) of both sexes. Pregnant women, individuals who were bedridden or unable to undergo anthropometric measurements and those unable to respond the questionnaire were not included in the study. The OpenEpi program was used for the sample calculation, considering the following parameters: reference population of 43.431 individuals, confidence level of 95%, expected prevalence for low muscle mass of 15% (8), predicted sample error of 3.0%, and design effect of 1.0. Addition of 10% was included to cover losses or refusals and 10% to control of confounding factors, resulting in a final sample of 697 adults. The sampling process was by double-stage clusters, with the census tracts as the first-stage units and the households as the second-stage units. A total of 30 census tracts were selected from the 99 existing in the urban area of Viçosa, and in each case the blocks were identified and numbered to specify the order to start work. The detailed methodology of the research is described in a previous publication (22).

The research project, which is the basis for this study, was approved by the Research Ethics Committee of the Federal University of Viçosa (Official Letter 02/2013). All participants signed the Informed Consent Term.

### Socio-demographic variables and leisure-time physical activity

A structured questionnaire was applied to the participants to obtain the variables age (20-29, 30-39, 40-49 and 50-59 years), skin color (white and non-white), and gender (female and male).

The International Physical Activity Questionnaire (IPAQ) was used to assess the level of leisure-time physical activity (LTPA) (23). LTPA was determined from the time spent with leisure physical activities in a normal week. Subjects who scored ≥ 150 minutes were classified as physically active and subjects who scored < 150 minutes as insufficiently active (24).

### Anthropometric variables and body composition

Weight and height were measured with participants using as little clothing as possible and barefoot. Height was measured using a stadiometer and weight was measured in a Tanita^®^ digital scale. BMI was calculated using the formula weight/height^2^ (1).

Body composition and its indices were evaluated and quantified by the DXA Lunar Prodigy Advance DXA System (GE Healthcare) model. All evaluations were performed by the same technician using the Incore Users Manual standard procedure.

The analyzed parameters were: total lean mass (TLM); fat free mass (FFM); appendicular lean mass (ALM), obtained by the sum of the lean mass of the arms and legs; total fat mass (FM) and truncal fat mass (TrFM), all expressed in kg. In addition, the percentage of total body fat (BF) was analyzed. With the ALM data, we obtained the skeletal mass index (SMI) relative to the height (SMI_height_: ALM/height^2^, given in kg/m^2^), as proposed by Baumgartner and cols. (8); SMI relative to weight (SMI_weight_: ALM/weight x 100, given in %) (11); and SMI relative to BMI (SMI_BMI_: ALM/BMI, given in kg/kg/m^2^) (10). The fat mass index (FMI: FM/height^2^, given in kg/m^2^) was also obtained (13); and the two load-capacity metabolic indices (LCMI) proposed by Siervo and cols. (2): LCMI_total_: FM/FFM e LCMI_regional_: TrFM/ALM.

### Statistical analyzes

The descriptive analysis was carried out using mean and standard deviation and estimates of absolute and relative frequency, after testing the normal distribution of the variables with the Shapiro-Wilk test, skewness coefficient, and graphical analysis. The Student’s *t* test was used to compare means of body composition and anthropometric variables between the sexes, and Pearson’s Chi-square test to compare frequencies of categorical variables between the sexes. Pearson’s correlation coefficient was used to evaluate the correlation between body composition indices and age. The level of significance for all tests was 5%. These analyses were performed with the statistical program Stata version 13.1.

To determine the cutoff point for lean mass deficit in the study population, data for young adults aged 20 to 39 years were used as a reference. In this subgroup, the value of two standard deviations (SD) below the mean was established as the lower limit for SMI_height_ (8) and SMI_BMI_ (10) and one SD below mean for SMI_weight_ (11).

Curves were constructed from the percentiles 20, 50, 75, and 90 of body composition indices, stratified by age group of 10 years and sex, using Excel (version 2010, Microsoft Office). It was decided to adjust a third degree polynomial function that promotes smoothing of the curves, suppressing small variations in its shape and allowing better visualization (25). The 50th percentiles of SMI_height_, FMI, and BF percentage were compared with reference values for the non-Hispanic and Mexican whites and blacks, derived from the GE Healthcare Lunar DXA system, published by the National Health and Nutrition Examination Survey (NHANES) 1999-2004 (21) as well as data of Chinese population (18). The 50^th^ percentile of LCMI_total_ and LCMI_regional_ were compared with secondary data from NHANES 1999-2004, but derived from the Hologic DXA system model without distinction of ethnicity (2).

## RESULTS

The study included 689 subjects, with mean age of 36.10 years (SD = 12.27 years).Women were significantly older than men. Most participants were not white and were physically inactive. A significant difference was found for anthropometric and body composition indices between the sexes. Men were taller, had higher body weight and higher values of total and regional lean mass, as well as higher values of the three SMI evaluated, whereas the women showed higher values for total and regional fat mass, as well as higher values of LCMI. Over 40% of the sample was in the overweight and obesity range (Table 1).

**Table 1:** Characteristics of study participants. Viçosa-MG, Brazil, 2012-2014 (n = 689)

Variables	Total (n = 689)	Men (n = 302)	Women (n = 387)	p value
Age (years)	36.10 (12.27)	34.46 (11.94)	37.39 (12.39)	<0.01
Skin Color				<0.05
White	287 (41.65)	141 (46.69)	146 (37.73)	
Non white	402 (58.35)	161 (53.31)	241 (62.27)	
LTPA				0.73
PA	203 (29.46)	91 (30.13)	112 (28.94)	
IA	486 (70.54)	211 (69.87)	275 (71.06)	
Weight (kg)	69.93 (14.54)	77.63 (13.20)	63.88 (12.57)	<0.001
Heighy (m)	1.66 (0.09)	1.74 (0.06)	1.60 (0.06)	<0.001
BMI (kg/m^2^)	25.10 (4.61)	25.31 (4.14)	24.94 (4.95)	0.28
TLM (kg)	44.91(10,44)	54.62 (6.86)	37.33 (5.12)	<0.001
ALM (kg)	20.08 (5,45)	25.14 (3,68)	16.12 (2.59)	<0.001
FM (kg)	22.49 (9.50)	19.94 (9.20)	24.48 (9.26)	<0.001
BF	31.70(10.02)	24.78 (8.29)	37.12 (7.64)	<0.001
FMI (kg/m^2^)	8.23 (3.66)	6.58 (3.05)	9.52 (3.57)	<0.001
SMI_height_ (kg/m^2^)	7.12 (1.34)	8.25 (0.95)	6.25 (0.86)	<0.001
SMI_weight_ (%)	28.79 (5.08)	33.01 (3.82)	25.49 (3.13)	<0.001
SMI_BMI_ (kg/kg/m^2^)	0.81 (0.21)	1.00 (0.15)	0.65 (0.10)	<0.001
LCMI_total_	0.49 (0.22)	0.34 (0.15)	0.61 (0.19)	<0.001
LCMI_regional_	0.63 (0.29)	0.46 (0.22)	0.76 (0.27)	<0.001
Nutritional status				* #<0.008
Low weight	22 (3.19)	7 (2.32)	15 (3.88)	
Normal weight	366 (53.12)	151 (50.00)*	215 (55.56)*	
Overweight	207 (30.04)	112 (37.09)*#	95 (24.55)*#	
Obesity	94 (13.64)	32 (10.60)#	62 (16.02)#	

Values presented in relative frequency (%) or mean (SD). SD: standard deviation; BMI: body mass index; TLM: total lean mass; ALM: appendicular lean mass; FM: total fat mass; FMI: fat mass index; SMI: skeletal mass index; LCMI: load-capacity metabolic index; LTPA: leisure time physical activity; PA: physically active; IA: insufficiently active.

* ^#^ Multiple comparison test.

The parameters evaluated showed a significant correlation with age. Correlation coefficients were similar between men and women. SMI_weight_ and SMI_BMI_ presented a negative correlation (-0.263 and -0.382 in males and -0.364 and -0.471 in females, respectively) and the other indices correlated positively with age (FMI: 0.265 and 0.334, BF: 0.228 and 0.293, LCMI_total_ 0.222 and 0.264, LCMI_regional_: 0.330 and 0.389, SMI_height_: 0.120 and 0.237 in men and women, respectively).

Cut-off points for SMI were generated in 204 men and 221 women between 20 and 39 years. The values found for SMI_height_ were 6.34 kg/m^2^ and 4.45 kg/m^2^, for SMI_BMI_ 0.74 kg/kg/m^2^ and 0.49 kg/kg/m^2^, and for SMI_weight_ 29.84% and 23.15%, respectively, for men and women. Our cut-off points were lower than those proposed by the other studies presented, except for the SMI_height_ value proposed by a study carried out in the Korean population (Table 2).

**Table 2:** Comparison of cut-off points proposed by different studies for skeletal mass indices

	Men	Women

	SMI_height_ (kg/m^2^)	
	
Current Study	6.34	4.45
Baumgartner (8)	7.26	5.45
Australian (19)	6.94	5.30
AWGS (28)	7.00	5.40
KHANES IV (27)	5.51	4.24

	**SMI_weight_ (%)**	

Current Study	29.84	23.15
KHANES (35)	30.98	24.81

	**SMI_BMI_ (kg/kg/m^2^)**	

Current Study	0.74	0.49
FNIH Sarcopenia Project (10)	0.78	0.51

SMI: skeletal mass index; AWGS: Asian Working Group for sarcopenia; KHANES: Korean National Health and Nutrition Examination Survey; FNIH: Foundation for the National Institutes of Health.

**Figure 1:**
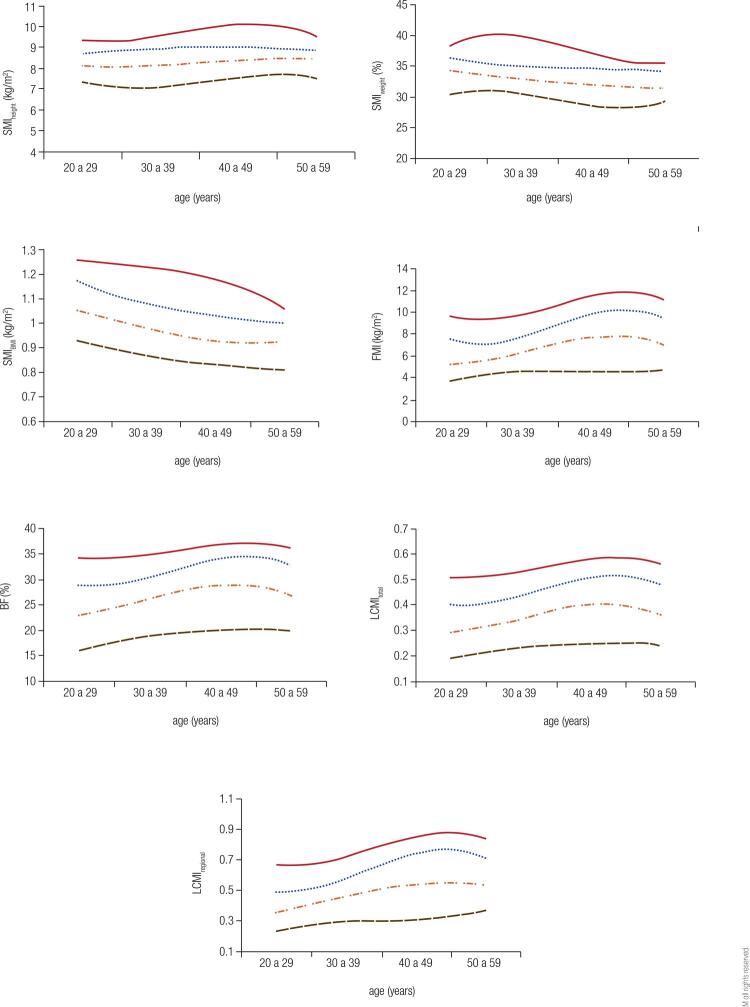
20, 50, 75 and 90 percentile curves for the indices evaluated among men. SMI: skeletal mass index; BMI: body mass index; BF: body fat; FMI: fat mass index; LCMI: load-capacity metabolic index.

**Figure 2:**
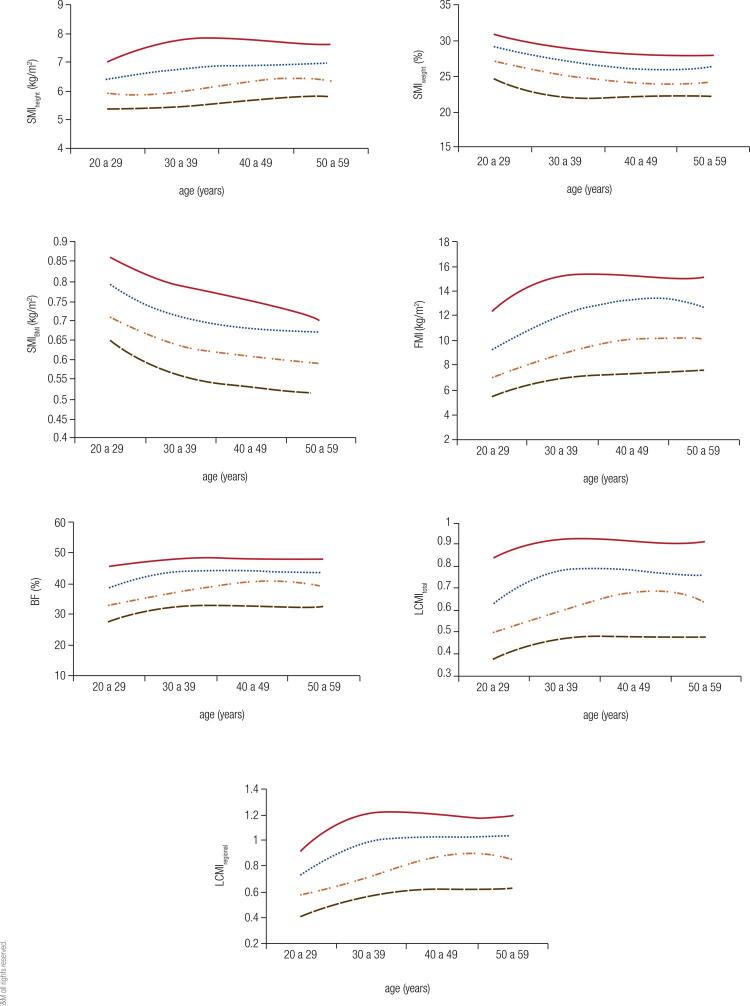
20, 50, 75 and 90 percentile curves for the indices evaluated among women. SMI: skeletal mass index; BMI: body mass index; BF: body fat; FMI: fat mass index; LCMI: load-capacity metabolic index.

Figures 1 and 2 show the percentile curves for the indices evaluated in men and women, respectively. In both sexes, the SMI_weight_ and SMI_BMI_ showed a decrease as early as 20-29 years, with a sharper descending slope visualized in the second index. SMI_height_ showed a sligth upward curve for both men and women. Only the 90th percentile curve showed a slight slope at 50-59 years. LCMI, in turn, increased up to the age of 40-49, when their curves stabilized for both sexes. Similarly, there was evolution of BF for men and women and FMI for women, with increase until the fifth decade and subsequent stabilization. For men, FMI peaked in the transition from the fifth to the sixth decade, with a downward trend thereafter.

The detailed values of the percentiles for the indices evaluated, according to age and sex, can be found in the supplementary material.

**Figure 3:**
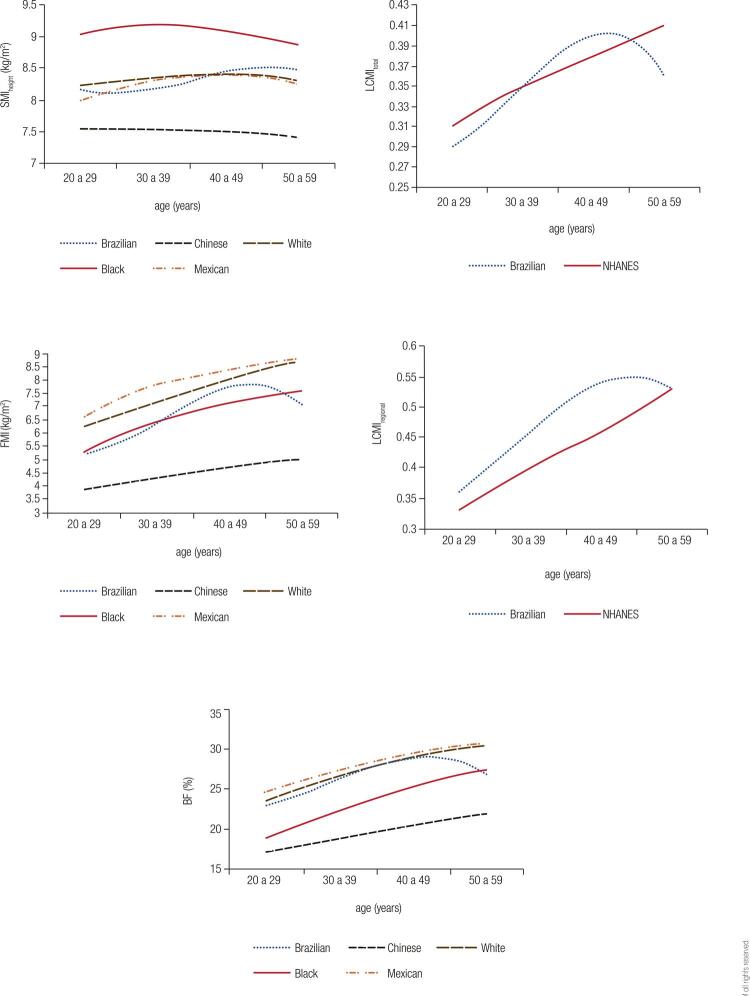
Comparison curves of the 50th percentile of different populations for men. SMI: skeletal mass index; FMI: fat mass index; LCMI: load-capacity metabolic index; BF: body fat.

**Figure 4:**
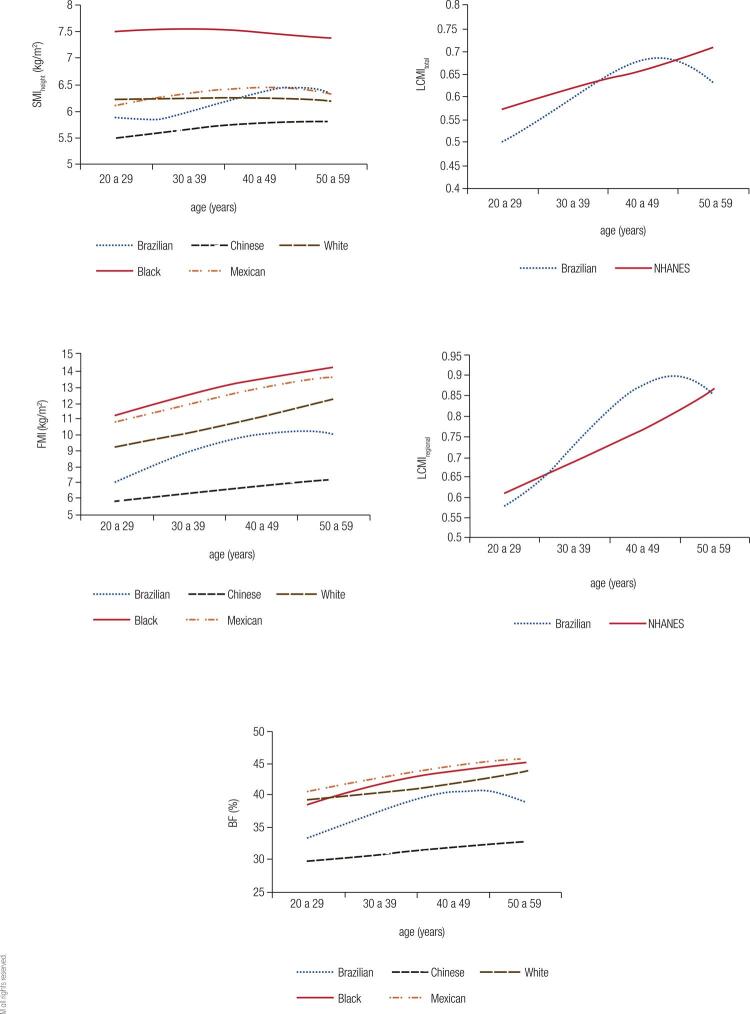
Comparison curves of the 50th percentile of different populations for women. SMI: skeletal mass index; FMI: fat mass index; LCMI: load-capacity metabolic index; BF: body fat.

When comparing our results on SMI_height_, FMI and BF with those from the Chinese and the NHANES (non-Hispanic whites and blacks and Mexicans) studies, we found that the Chinese had the lowest values for all age groups and both genders. Blacks presented the highest values for SMI_height_, and our data were similar to those of whites and Mexicans for both men and women. Our representative SMI_height_ curves showed a peak followed by stability in the fifth decade of life, while the other ethnicities generally peaked between 30-39 years, with a slight fall from 40-49 years, which was more evident among men (Figures 3 and 4).

White and Mexican men had the highest FMI, and our sample showed similar values to those of blacks up to 30-39 years, but then, our sample surpassed the latter, peaking at 40-49 years and then falling. This fact was not recorded in the other ethnic groups, which maintained their ascending curves (Figure 3). Among women, who presented slightly ascending curves in practically all age groups, our values were intermediate between whites and Chinese with a slight decline at the beginning of the sixth decade. Non-Hispanic black women and Mexican women had the largest FMI (Figure 4).

BF of males of our population, of non-Hispanic whites, and Mexicans were the highest and similar among them, but after the 40-49 years, our data dropped, while the other curves maintained growth (Figure 3). In turn, women of the three ethnicities from the NHANES study showed higher BF, with the results of our sample approaching them at 40-49 years, but falling at 50-59 years. As with the male curves, the other ethnicities maintained the growth pattern (Figure 4).

The LCMI curves of our sample, for both sexes, peaked at 40-49 years and fell at 50-59 years, unlike the Americans, whose curves showed constant growth at all ages. LCMI_regional_ of our sample was higher than those of the Americans for most of the age groups evaluated. LCMI_total_, in contrast, was superior to that of Americans only between 40-49 years (Figures 3 and 4).

## DISCUSSION

To our knowledge, this is the first Brazilian population-based study to evaluate percentile curves for SMI, LCMI, FMI and BF in adult population.

We found significant differences between sexes for the various body composition data. Women had higher adiposity and man had higher values of lean mass, a fact already observed in other ethnic groups (15,18,20,26). BMI was similar between the sexes, confirming the weakness of this index as a measure of body composition (4).

Pearson correlation coefficients in both sexes revealed significant associations between the indices studied and age, with SMI_BMI_ and LCMI_regional_ showing the strongest correlations. SMI, with the exception of SMI_height_, had negative correlations with age and FMI and LCMI had positive correlations, findings that emphasize the importance of studying the evolution of these indices throughout the lifespan. The negative correlation between age and the index proposed by Baumgartner and cols. (8) was not confirmed in our sample, differing from most of the authors. This result may be due to the fact that the other studies included, for the most part, elderly individuals (18-20,27). SMI_height_ does not consider fat mass in its adjustment from ALM, overestimating lean mass in overweight or obese individuals (6). Thus, in adulthood, increased adiposity may erroneously reflect an increase in SMI_height_.

The comparison of the SMI cutoff points indicated variation among the different ethnic groups. In both, men and women, the cut-off points defined in our sample were the lowest for the three indices, the only exception being the cut-off point of Koreans for SMI_height_, which was the lowest among the ethnicities evaluated and corroborates the findings of lower muscle mass in this population (27).

The low cut-off point of our sample in the three lean mass indices studied may be related to the high prevalence of leisure-time physical inactivity (about 70% in the sample). This finding may be a reflection of the urbanization process of the last decades, which is characterized by sedentary lifestyle, a diet rich in low nutritional value foods, and with high caloric value. This fact was also reported in an Asian study, with especially low cut-off points (28). A study with Brazilian men evaluated the cut point of SMI_height_ and found the value of 6.6 kg/m^2^. However, the reference population was in the age range of 20-29 years, which limits the comparison with our results (29). The difficulty in comparing with other national studies is related to different methodologies applied, such as the use of bioimpedance and predictive equations, as well as the use of different age groups (30-32).

When we evaluated the percentile curves of SMI in our sample, SMI_height_ was not able to identify lean mass loss in the studied ages, whereas SMI_weight_ and SMI_BMI_ detected a decline from the third decade of life between men and women. Since approximately 43% of our sample is in the overweight or obesity range, the lean mass loss assessed by the SMI_height_ may have been underestimated, corroborating the hypothesis that increased adiposity may reflect an increase in the lean mass when using this index (6,16). Most studies evaluating the percentiles of body composition indices (15,18-21,33) used the Baumgartner and cols. definition (8), while data with SMI adjusted for body weight and BMI were scarce. In a study with Brazilians above 20 years of age, Ushida and cols.(29) and Sousa and cols. (33) found that SMI_BMI_ decreased after 50 years in men, whereas using SMI_height_, this decrease occurred later, at 60-69 years in men and 50-59 years in women. When we evaluated the comparative curves of SMI_height_ obtained in our study, we found that both Chinese and Americans of both sexes showed a slight decline after 40-49 years, while our sample showed growth from 30-39 years until stabilization at the end of the next decade. In other populations, such as Australia and Mexico, where SMI_height_ was used, it was possible to detect the decline in lean mass from the fifth or sixth decade of life in men and women, respectively (19,20). But in these studies, the largest declines occurred after age of 70 years and were more pronounced and accelerated in men.

In the present analysis, we used the LCMI proposed by Siervo and cols. (2) that fill a gap in the studies of body composition, which is the identification of individuals with both high adiposity and low muscle mass (2,17). It is a model that demonstrates the metabolic imbalance in the individual, that is, refers to the tissues that threaten the homeostasis of the organism and to those that maintain it, respectively, fat and musculature (2). The women presented the highest mean LCMI, but we found that both sexes evolved with an increase in these indices until the fifth decade of life, when they began a slight decline. In the NHANES study, this peak occurred between 60-70 years, and until that age, there was a permanent growth with later decline in comparison with our sample, showing the increasing degree of metabolic imbalance of Americans throughout adulthood (2). However, when assessing LCMI_regional_, our data on men and women remained superior to those of Americans throughout virtually the entire adulthood, despite the decline that began in the sixth decade. This regional index relates TrFM to ALM, referring to adiposity, mainly visceral or android, which is associated with high morbidity and mortality due to cardiovascular diseases in adults (34). These findings may reflect the high physical inactivity of the sample and warn about poor eating habits and the need for early measures of cardiometabolic risk prevention in this age group.

Data related to BF and FMI show the peak of adiposity between 40-49 years in men and women, but with a better scenario when compared to the Chinese and American population, where growth is maintained beyond the sixth decade, confirming the concern with the global growth of obesity (1,27). Studies with the American population indicate the increase in adiposity up to 80 years in men and 65 years in women (26), and this sustained increase is also observed among Asians despite their lower values of fat mass (18,27).

Some limitations of our study should be considered. The size of the sample used limits stratified analyses according to other characteristics such as ethnicity or skin color. The scarcity of data converted to the GE Lunar system, especially with regard to LCMIs, limits the conclusions based on comparisons of these indices. As a strength of our work, we highlight the sampling process, which allowed for the first time in Brazil a population-based study approaching body composition indices using percentile curves. Although our study was carried out with a representative sample of adults from a city of Minas Gerais, caution should be taken in extrapolating the results to the whole Brazilian population, taking into account the size of our country, the large population and the different characteristics of each region.

In conclusion, the data and curves showed that the SMI adjusted for BMI and body weight were more adequate in detecting the decline of lean mass in adults due to aging. In contrast, SMI_height_ had a positive correlation with age and its curve increased throughout the evaluated age groups.

LCMI curves and adiposity measurements showed growth up to the fifth decade of life, demonstrating the pattern of increasing metabolic imbalance of our sample in adulthood. Americans and Chinese maintained an upward curve until the sixth decade of life. The LCMI_regional_ of our sample, which was superior to that of the Americans in practically all the adult age, reflects the early growth of visceral adiposity in the study population.

The data presented in this study contribute to the individual and collective assessment of the nutritional status of adults and also to the prevention, early identification, and treatment of outcomes related to adiposity and lean mass deficit. In addition, these data will be useful for comparison with future epidemiological studies.
